# Multimodal therapy for synergic inhibition of tumour cell invasion and tumour-induced angiogenesis

**DOI:** 10.1186/1471-2407-10-92

**Published:** 2010-03-11

**Authors:** Pamela Zengel, Diana Ramp, Brigitte Mack, Stefan Zahler, Alexander Berghaus, Bernd Muehlenweg, Olivier Gires, Suna Schmitz

**Affiliations:** 1 Department of Otorhinolaryngology, Head and Neck Surgery, Grosshadern Medical Center, Ludwig-Maximilians-University of Munich, Marchioninistr 15, 81377 Munich, Germany; 2 Department of Inner Medicin, Zentral Klinikum Augsburg, Strenglinstrase 2 86156 Augsburg, Germany; 3 Department Pharmazie-Zentrum für Pharmaforschung -, Ludwig-Maximilians-Universität München, Butenandtstr. 5-13, Haus B, D-81377 Munich, Germany; 4 Wilex AG München, Grillparzerstrasse 10, D-81675 Munich, Germany; 5 Clinical Cooperation Group Molecular Oncology, Helmholtz-Zentrum München, German Research Center for Environmental Health, and Head and Neck Research Dept, Ludwig-Maximilians-University of Munich, Germany; 6 Amper Kliniken AG, Abt für Hals-Nasen-Ohrenheilkunde, Krankenhausstr 15, D-85221 Dachau, Germany

## Abstract

**Background:**

Squamous cell carcinoma of the head and neck (SCCHN) are highly invasive tumours with frequent local and distant recurrence. Metastasis formation requires degradation of the extracellular matrix, which is fulfilled by membrane-associated proteases such as the urokinase plasminogen activator (uPA). WX-UK1 is a competitive active site inhibitor of the protease function of uPA that impairs on the capacity of tumour cells to invade *in vitro*.

**Methods:**

In the present study, effects of combinations of WX-UK1 with matrix metalloprotease inhibitors (MMP, galardin^®^) and cyclooxygenase-2 (COX-2, celecoxib^®^) inhibitors on tumour cell proliferation, invasion, and angiogenesis induction were evaluated. Matrigel invasion chambers and a spheroid co-cultivation model with human fibroblast served to determine the invasive potential of both FaDu (SCCHN) and HeLa (cervical carcinoma) cells, each treated with combinations of Celecoxib^®^, Galardin^®^, and WX-UK1.

**Results:**

Blocking of single protease systems resulted in a significant 50% reduction of tumour cell invasion using WX-UK1, while the triple combination was even more effective with 80% reduction of invasion. Additionally, a sprouting assay with HUVEC was used to test the anti-angiogenetic potential of the triple combination, resulting in a 40% decrease in the sprouting rate.

**Conclusions:**

A combined approach targeting different families of proteases and cyclooxygenases represents a promising adjuvant therapy.

## Background

Squamous cell carcinoma of the head and neck (SCCHN) are aggressive tumours, which are still associated with poor prognosis despite improvements in surgical and radiotherapeutic techniques [1]. SCCHNs exhibit highly invasive growth, aggressive metastasis formation, and early recurrence [1]. In order to metastasise, tumour cells utilize a complex set of molecular mechanisms [2]. Migration through surrounding tissue is achieved upon the degradation of the extracellular matrix (ECM) by both, membrane-fixed and soluble proteases. In this respect, matrix metalloproteases (MMP) and the urokinase-type plasminogen-activator system (uPA), which is responsible for the conversion of plasminogen into plasmin, are of major importance. The activity of MMPs and uPA fosters cell migration, angiogenesis and metastasis [3,4]. Tumours greater than 1.5 mm^3 ^in size are strictly dependent on intimate contacts to blood vessels or otherwise become necrotic [5]. Neo-angiogenesis is provided *via *the production of growth factors such as basic fibroblast growth factor (bFGF) and vascular endothelial growth factor (VEGF), which attract endothelial cells into the tumour tissue to enable tumour progression. In turn, *de novo *formed vessels strengthen tumour invasion and metastasis through the production of MMP 2 and 9 and uPA, which further degrade ECM. The *in vivo *anti-metastatic and anti-proliferative activity of the synthetic uPA inhibitor WK-UK1 has been demonstrated in various animal tumour models. WX-UK1 is a serine protease inhibitor that inhibits protease upon attachment to the active center of the enzyme, resulting in a reduction of tumour expansion [6]. As the plasminogen activator system plays a role in coagulation, thrombotic vessel occlusion could be a risk during therapy, but these side-effects were neither observed *in vivo *nor *in vitro *[7]. MMPs are zinc-bound enzymes that degrade ECM and, particularly MMP 3, 2 and 9, play a role in tumour expansion, invasion and metastasis. According to the frequent over-expression of MMPs in malignant tumours and to the correlation with a highly aggressive phenotype and poor prognosis [8,9], inhibition of MMPs has provided a significant increase in the survival rate in clinical trials [10]. Combinatorial inhibition of MMPs and the plasminogen activator system using siRNA approaches likewise revealed efficient with a 60% and 90% down-regulation of invasion and angiogenesis, respectively [11,12].

Another group of anti-angiogenic active pharmaceutical agents comprises non-steroidal anti-inflammatory drugs. *In vitro *studies have suggested a potential of cyclooxigenase-2 (COX-2) inhibitors to block angiogenesis and reduce the growth of colon, head and neck, and skin tumours [13,14]. These therapy forms have fewer side effects in comparison to conventional treatment regimens such as chemotherapy and/or radiotherapy most probably owing to the fact that healthy persons utilize angiogenesis primarily to circumvent chronic inflammation diseases and during the female menstrual cycle. Importantly, resistance did not emerge in mouse models of fibrosarcoma, Lewis lung carcinoma and melanoma, after repeated *in vivo *treatment with the antiangiogenic agent endostatin [15,16]. The over-expression of components of the uPa system, pro-angiogenic cytokines such as bFGF and VEGF, and MMPs in malignant tumours in comparison to normal tissue, correlates with a poor prognosis and a higher recurrence rate [8].

Numerous synthetic protease inhibitors have been tested in clinical trials. Our own group described a 50% inhibition of tumour cell invasion using the serine protease inhibitor WK-UK1 *in vitro *[17], however a wide clinical application as a monotherapy has not been put into practice yet. Previous expertise disclosed that only multimodal therapy strategies take into account the plethora of mechanisms underlying tumour progression and are hence indispensible. Thus, a combination of inhibitors that address different aspects of tumour progression and metastasis formation is a promising concept [18,19].

In this study, the serine protease inhibitor WK-UK1, the MMP inhibitor Galardin^® ^and the selective COX-2 inhibitor Celecoxib^® ^have been tested as a combinatorial treatment with chemical compounds against carcinoma cells. Treatment of carcinoma cells resulted in a significantly improved inhibition of invasion as compared to monotherapy with WX-UK1 and sprouting of the endothelial cells was inhibited by about 40%, too. Thus, our results suggest that the combination of three classes of inhibitors is potently decreasing metastatic spread and neo-angiogenesis.

## Methods

### Cell lines

FaDu (SCCHN), HeLa (cervical carcinoma), and human primary skin fibroblasts (generated from skin biopsies from healthy volunteer donors) were cultured in standard Dulbecco's Modified Eagle Medium (DMEM) containing 10% fetal bovine serum (FCS), with 1% penicillin-streptomycin and 1% sodium pyruvate, in a humidified incubator at 37°C at 5% CO_2 _to confluence. HUVEC were freshly isolated from human umbilical veins of newborns by collagenase digestion, as described previously [20]. HUVECs were grown in monolayers, were harvested by centrifugation and amplified at 37°C in endothelial cell growth medium (EBM2, Cambrex, Verviers, Belgium). In all our experiments, only the first three passages of each HUVEC primary culture were used. Single-cell suspensions were performed by mild enzymatic dissociation using trypsin/EDTA (0.05%/0.02% w/v) solution in PBS.

### MTT assay

Cellular metabolism was assessed in a standard MTT conversion assay as described previously [21]. Briefly, FaDu, HeLa, fibroblasts and HUVE cells (3 × 10^3 ^cells/well) were plated in 96-well plates and analyzed at the time points indicated.

### Immunostaining of chamber slides

For immunohistochemical staining, chamber slide cultures (Quadriperm, Sigma Aldrich) were generated from human epitheloid carcinoma cell lines FaDu and HeLa using 4-5 × 10^5 ^cells per slide. Immunodetection of MMP 1, 2, 9 (IgG mouse anti human, 1:100; R&D System, Wiesbaden, Germany), MMP 3 (IgG goat anti human, 1:100; R&D System, Wiesbaden, Germany), COX-2 (IgG rabbit anti human, Medac, Wedel, Germany) and VEGF (IgG goat anti human, 1:100; R&D System, Wiesbaden, Germany) in chamber slides was performed using the standard ABC method as explained below. The specific staining was visualized in red and slides were counterstained with hematoxilin, giving blue colored nuclei.

Negative control staining for immunohistochemistry was performed in the absence of primary antibody.

### Spheroid co-culture model

A spheroid co-culture model was established according to the method described by Kunz-Shughart et al. [22], in order to assess the invasive potential of FaDu and HeLa cell lines into fibroblast spheroids. In order to best imitate minimal residual disease, single-cell tumour suspensions were added pre-formed fibroblast spheroids, which were generated on 96-well plates (100 μl/well) coated with 1% agarose. For this purpose, primary fibroblasts (1 × 10^4^) were plated separately in 100 μl DMEM (10% FCS) per well. After 24 hours, single cell suspensions (FaDu 3 × 10^3^, HeLa 8 × 10^3^) were incubated for further 3 days, renewing the medium every 48 hours. WX-UK1, Celecoxib^® ^and Galardin^® ^were added to culture media every 48 hours in the concentrations described below, as single agents or in double and triple combinations. Spheroid specimens were embedded in tissue-tek (Sakura Finetek, Torrance, CA), shock-frozen in liquid nitrogen and stored at -20°C. This process was carried out in 3 independent experiments, each resulting in 6-8 single co-culture samples.

### Immunohistochemistry (double staining)

Multiple cryosections (4 μm) of each spheroid co-culture specimen were fixed in acetone (10 min, RT) and incubated in H_2_O_2 _(10 min, RT, 0.03%) to block endogenous peroxidase activity. After washing in PBS, slides were incubated with either EpCAM-specific MAb C215 (1:200, kind gift of Dr. H. Lindhofer, GSF, Munich, Germany) or the cytokeratin-specific MAb KL1 (1:500; pan-cytokeratin, reacts with several cytokeratin subtypes; Coulter-Immunotech Diagnostics, Krefeld, Germany). The standard ABC kit (Vectastain; Vector, Burlingame, CA) was used to detect for an Antigen-antibody reaction. The peroxidase reaction was developed with AEC as a chromogen (Sigma, St. Louis, MO), resulting in red staining. The additional prolyl-4-hydroxylase-specific MAb 5B5 (1:100; Dako, Glostrup, Denmark) was visualized by the APAAP method. After incubation with MAb 5B5, sections were incubated with goat antimouse IgG (1:25) and mouse APAAP complex (1:50; both from Dako). Finally, staining was performed with fast blue BB salt (Sigma), and sections were mounted in Kaiser's glycerol gelatin for subsequent analysis. Negative control staining for immunohistochemistry was performed in the absence of primary antibody.

### Evaluation of immunostaining

The invasive potential of tumour cells into pre-formed fibroblast spheroids was quantified as follows: quadrants invaded by tumour cells were counted by two experienced investigators independently, and divided by all counted quadrants of the spheroid. Invasion proportion was calculated as the proportion of fibroblast quadrants invaded by tumour cells relative to control-treated cells.

### Matrigel invasion assay

The invasion potential of cells was assessed in a matrigel invasion chamber using the BD BioCoat Matrigel Invasion Chamber (Becton Dickinson Biosciences, Bedford, MA). Briefly, 750 μl of NIH-3T3 conditioned fibroblast supernatant or alternatively from primary human fibroblasts derived from a hypopharynx carcinoma (24 hours in serum-free medium) were added to wells of the companion plate, and a cell suspension containing 2.5 × 10^4 ^FaDu or HeLa tumour cells in 500 μl medium (0.1% FCS) was added into inserts for 24 hours, as was WX-UK1 (Wilex, Munich, Germany) at a concentration of 1.0 μg/ml, Galardin^® ^(5 M/μl, Calbiochem, Germany), and Celecoxib^® ^(5 μM/ml, Molekula Nienburg, Weser).

Transmigrating cells were stained with toluidine blue and counted at a 320-fold magnification by light microscopy. The four quarters of the membrane (0.8 cm diameter) were counted separately, each corresponding to one visual field under the microscope, where the margin containing remaining cells that could not be rinsed out was not considered. Three individual experiments with each cell line were performed.

### Migration assay

The migration of cultured FaDu, HeLa and HUVECs in the absence and presence of inhibitors was assayed by using transwell chamber with 8 μm pores (Corning Costa, Cambridge, Ma). Cultured cells were trypsinized and suspended at a concentration of 1 × 10^6 ^cells/ml. 100 μl of this suspension was placed on the upper chamber and treated with or without inhibitors. Conditioned supernatant of murine NIH-3T3 fibroblast (24 hr in serum-free medium) was added to wells of the companion plate as a chemoattractant. The chamber was then incubated at 37°C for 8 hours, filters were removed, fixed and stained with toluidine blue, and counted at 320-fold magnification by light microscopy. The four quarters of the membrane (0.8 cm diameter) were counted separately, each corresponding to one visual field under the microscope, where the margin containing remaining cells that could not be rinsed was not considered. Three individual experiments with each cell line were performed allowing the calculation of ratios of Matrigel invasion/migration capacity.

### *In vitro *angiogenesis assay

HUVEC spheroids, each containing 1000 cells, were generated over night and embedded into collagen gels. A collagen stock solution was prepared prior to use by mixing acidic collagen extract of rat tails (equilibrated to 2 mg/ml, 4°C; 8 vol.) with 10× EBSS (Gibco BRL, Eggenstein, Germany; 1 vol.) and in approx. 1 volume of 0.1 N NaOH to adjust the pH to 7.4. This stock solution (0.5 ml) was mixed with 0.5 ml ECGM basal medium (PromoCell, Heidelberg, Germany) with 10% FCS, (Biochrom, Berlin, Germany) containing 0.5% (w/v) carboxymethylcellulose to prevent sedimentation of spheroids prior to polymerization of the collagen gel. Subsequently, 50-70 HUVEC spheroids and test substances were added to the mixture. The spheroid-containing gel was rapidly transferred into pre-warmed 8 well plates (Ibidi, Munich, Germany) and allowed to polymerize (1 min). Gels were incubated at 37°C in 5% CO_2 _atmosphere at 100% humidity. After 24 hours in-gel angiogenesis was quantified for each experimental group by using a light microscope to count the number of capillary-like sprouts of at least 10 spheroids. Three individual experiments were performed.

### Chemotactic migration assay

Chemotactic migration of cultured HUVEC was assayed using a Transwell chamber with 8 μm pores (Corning Costa, Cambridge, MA). Cultured cells, which were hungered over night, were trypsinized and suspended at a density of 1 × 10^6 ^cells/ml, and 100 μl was placed on the upper chamber. As a chemotactic agent, the lower chamber was filled with 500 μl of conditioned supernatant of treated or untreated tumour cells. The chamber was incubated at 37°C for 8 hours before removal of filters, fixation, staining with toluidine blue, and counting at 320-fold magnification by light microscopy. The 4 quarters of the membrane (0.8 cm diameter) were each counted separately as each corresponds to one visual field under the microscope. The margin containing the remaining cells that could not be rinsed was not considered. Three individual experiments were performed.

### ELISA

Concentrations of bFGF were assessed upon ELISA according to the manufacturer's protocol (Human FGF basic, R&D Systems, Mineapolis, MN, USA).

### Statistical evaluation

Significance of experimental data was calculated using a paired Student's *t*-test and the Excel software (Microsoft Corp., Redmond, WA) or a combination of ANOVA and Kruskal-Wallace tests. P-values are given when appropriate in the according figures.

## Results

### Triple inhibition of uPA, MMPs, and COX-2 decreases the invasive capacity of tumour cells

**Figure 1:**
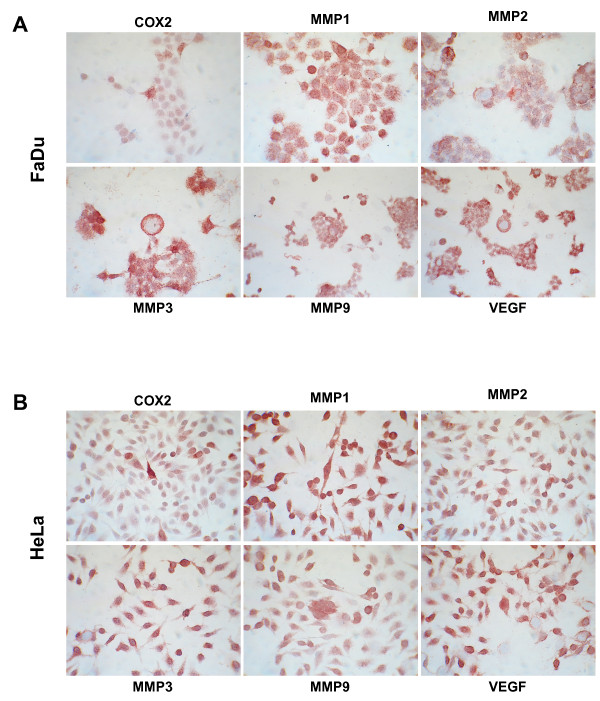
**MMP, COX-2 and VEGF expression**. Immunohistochemical detection of MMP 1, 2, 3, 9, COX-2 and VEGF in chamber slides of FaDu (A) and HeLa cells (B). Shown is one representative experiment out of three.

Inhibition of the uPA protease system with WX-UK1 displayed excellent results with a 50% rate of inhibition of tumour cell invasion into matrigel and in a spheroid co-culture model [17]. Nonetheless, this rate has room for improvement for example upon combinations of therapeutics. We chose three mechanisms contributing to tumour cells invasion and neo-angiogenesis to be inhibited in a multimodal fashion, *i.e. *uPA, matrix metalloproteases, and cyclooxygenase 2. The availability of targets was assessed in the SCCHN FaDu and in the cervix HeLa carcinoma cell lines. Expression of COX-2, MMP1-3 and 9, and VEGF was assessed *via *immunohistochemistry with specific antibodies and revealed intermediate (COX-2) to high (Figure 1). Expression patterns of uPA and uPA-R have been displayed in our former studies and both cell lines revealed positive for components of the uPA-system [17]. Potential toxic effects of each compound as a single and multimodal treatment were assessed in a standard vitality MTT assay. No significant decrease or increase in cell vitality was observed upon treatment of FaDu and HeLa cells with all three compounds (additional file 1).

**Figure 2:**
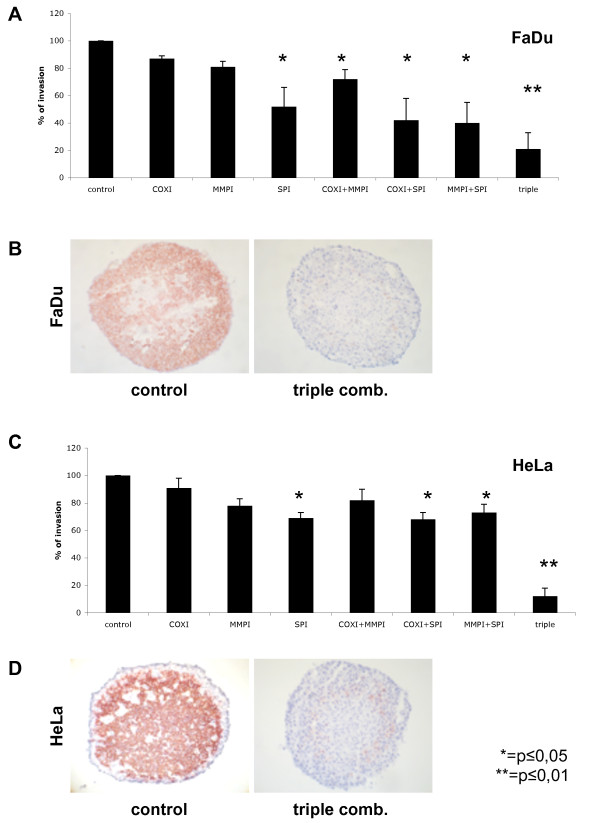
**Effects of WXUK-1 (SPI), Celecoxib (COXI), and Galardin (MMPI) on FaDu and HeLa cell invasion capacity**. (**A**) FaDu cells (3000 cells) or (**C**) HeLa cells (8000) were cultured in absence or presence of single, double or triple combination therapy. Shown is the mean infiltration of tumour cells with standard deviation into 15-18 pre-formed fibroblast spheroids performed in three independent experiments. (**B**) FaDu and (**C**) HeLa single cells (brown staining) and human primary skin fibroblast spheroids (1 × 10^4 ^cells, blue staining) were visualised by immunohistochemistry with antibodies against cytokeratines and MAb 5B5 in kryosections at day three of co-culture in the absence (left sections) and in the presence of inhibitors (right sections).

Next we generated spheroids from primary skin fibroblasts and incubated pre-formed spheroids with single cell suspensions of FaDu or HeLa cells. Invasion of pre-formed fibroblast spheroids by single tumour cells was monitored in cryosections and after staining of tumour cells with cytokeratin-specific antibodies as described in materials and methods. The nature of invading cells was further corroborated upon staining for the tumour-associated antigen EpCAM [23] (data not shown). Untreated FaDu and HeLa cells were characterised by a strong invasion capacity into fibroblast spheroids, which was set to 100% for the purpose of comparison (Figure 2). Next, FaDu and HeLa cells were treated with Celecoxib^®^, Galardin^®^, WX-UK1, and combinations thereof, before incubation with fibroblast spheroids. Celecoxib^®^, Galardin^® ^displayed no major effect on tumour cell invasion when applied as single drugs and at the concentration chosen, while the combination of both inhibitors resulted in a 30% reduction for FaDu cells (Figure 2A and 2C). WX-UK1 inhibited tumour cell invasion by 50% and 30% in FaDu and HeLa cells, respectively (Figure 2A and 2C). However, the most effective inhibition was achieved with a triple combination of WX-UK1, Galardin^®^, and Celecoxib^® ^with 80% and 90% of inhibition for FaDu and HeLa cells, respectively (Figure 2).

**Figure 3:**
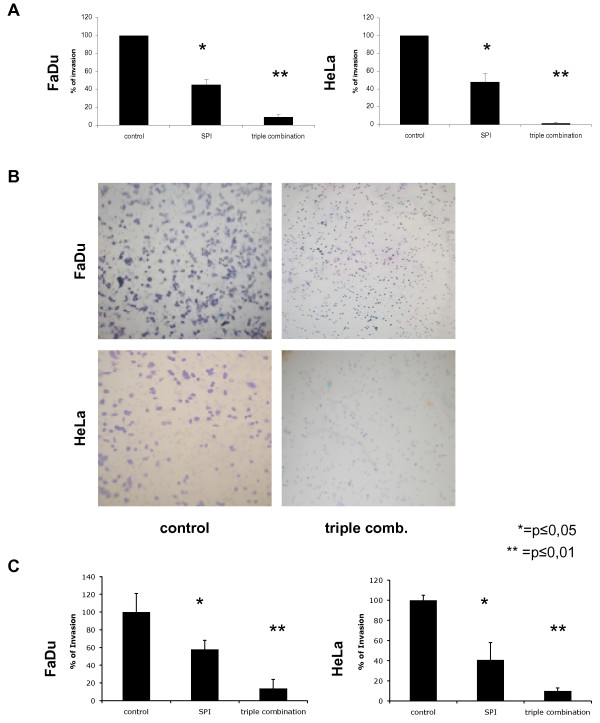
**Effects of WX-UK1, Galaradin^®^, and Celecoxib^® ^on matrigel transmigration of tumour cells**. (**A**) Shown are mean of invasion/migration ratios with standard deviation from three independent experiments. FaDu and HeLa cells were treated with WK-UK1^® ^(SPI), triple combination of WX-UK1, Galaradin^®^, and Celecoxib^®^, or kept untreated (control). (**B**) Representative images of FaDu and HeLa cells after matrigel transmigration in the absence (left panels) or presence of WX-UK1, Galaradin^®^, and Celecoxib^® ^are shown (right panels). (**C**) Same experiments as in (A) with supernatants from primary fibroblasts derived from hypopharynx carcinoma samples. Shown are mean of invasion/migration ratios with standard deviation from three independent experiments.

Fibroblast spheroids emulate the tissue surrounding tumours and metastasis and hence represent a valuable model for invasion and tissue remodelling. However, in order to obtain a more direct evidence for the effect of medication on the invasion capacity of tumour cells, transmigration experiments with matrigel invasion chambers were conducted. FaDu and HeLa cells were treated with WX-UK1 alone or a combination of WX-UK1, Galardin^®^, and Celecoxib^®^, and plated on matrigel invasion chambers composed of 6-well inlays with 8 μm pores coated with extracellular matrix (ECM) and allowed to transmigrate towards murine NIH3T3 fibroblasts' supernatants. As a control, transmigration values were related to the migration capacity of cells through inlays lacking the ECM coat. Inhibition of the uPA system with WX-UK1 alone resulted in a 55% reduction of transmigration of FaDu and HeLa cells as compared with mock treated cells. Incubation of cells with a triple combination yielded an average 90-95% inhibition of transmigration (Figure 3A and 3B). In order to strengthen the correlation to the situation *in vivo*, we conducted additional transmigration experiments with supernatants from human, primary fibroblasts derived from head and neck carcinoma samples. The results obtained herewith were similar to experiments performed with NIH3T3 supernatants and corroborated the efficacy of triple medication on the invasive capacity of FaDu and HeLa tumour cells (Figure 3C).

### COX-2, MMPs, and uPA-system inhibition impacts on neo-angiogenic potential of tumour cells

**Figure 4:**
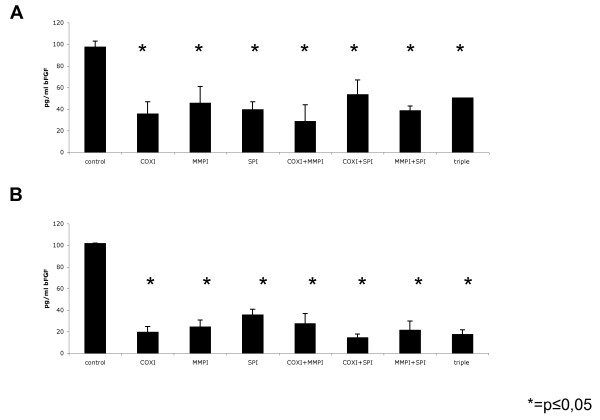
**Amounts of bFGF in supernatants of FaDu and HeLa cells measured *via *ELISA, treated with WX-UK1, Galardin^®^, and Celecoxib^® ^as single drugs and with combinations thereof**. The COX-2 inhibitor Celecoxib^® ^displayed the greatest effects as a single drug and in combination with WX-UK1 and/or Galardin^®^. Triple medication was neither for FaDu nor for HeLa cells more effective than Celecoxib^® ^alone, pinpointing that COX-2 inhibition was instrumental in the reduction of bFGF release by tumour cells.

**Figure 5:**
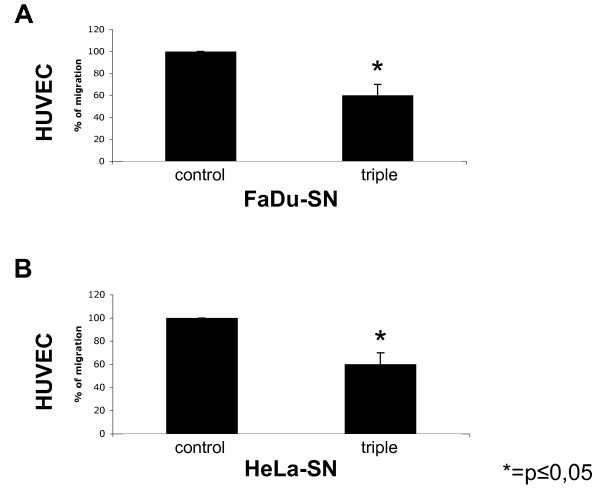
**Chemotactic potential of FaDu and HeLa cells on HUVEC**. Using the Boyden chamber assay the chemotaxis of HUVEC was assessed using supernatant of FaDu (A) and HeLa (B) cells kept untreated (left columns) or treated with the triple combination (right columns p < 0.01). Shown are mean percentages of migration with standard deviation of three independent experiments.

The ability of carcinoma cells to attract endothelial cells in order to generate new vessels is mandatory to support tumour maintenance and growth. Hence, we first analysed the effect of WX-UK1, Galardin^®^, and Celecoxib^® ^on the ability of FaDu and HeLa carcinoma cells to produce neo-angiogenic factors such as bFGF. FaDu and HeLa cells were treated with WX-UK1, Galardin^®^, and Celecoxib^® ^as single drugs and with combinations thereof, and thereafter amounts of bFGF in supernatants were measured *via *ELISA. The COX-2 inhibitor Celecoxib^® ^displayed the greatest effects as a single drug and in combination with WX-UK1 and/or Galardin^® ^(Figure 4). Triple medication was neither for FaDu nor for HeLa cells more effective than Celecoxib^® ^alone, pinpointing that COX-2 inhibition was instrumental in the reduction of bFGF release by tumour cells. Next, human umbilical vein endothelial cells (HUVEC) were used to monitor the chemoattractant potential of tumour cells depending on the treatment with WX-UK1, Galardin^®^, and Celecoxib^®^. FaDu and HeLa cells were kept untreated or incubated with a triple combination of WX-UK1, Galardin^®^, and Celecoxib^®^. Conditioned supernatants of these cells thereafter served as chemoattractants in the lower chamber of a transwell migration assay for HUVEC. Treatment of FaDu and HeLa cells resulted in a reduction of attraction of HUVEC by 40% in average (Figure 5).

**Figure 6:**
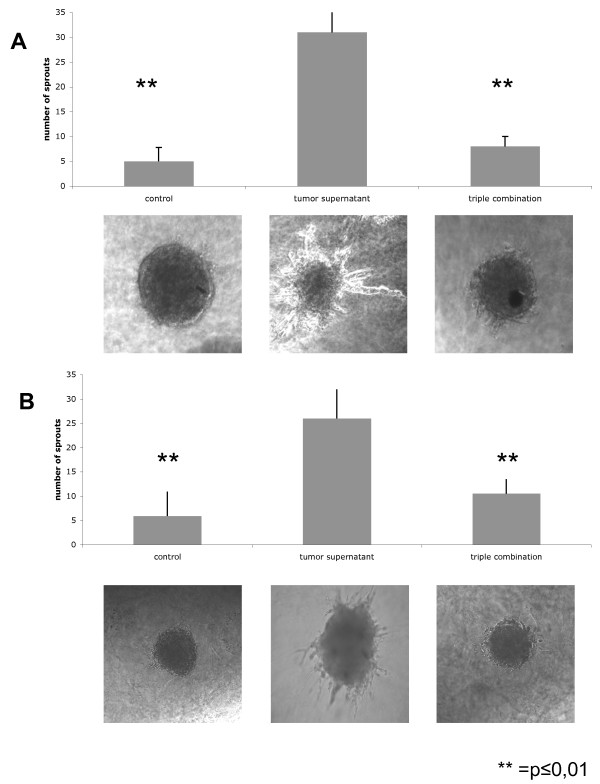
**Effects of WX-UK1 (SPI), Celecoxib^® ^(COXI), and Galardin^® ^(MMPI) on the sprouting capacity of HUVEC spheroids in collagen gel**. Shown are the mean numbers of sprouts of HUVEC with standard deviation from 15-20 individual spheroids performed in three independent experiments. HUVEC were incubated with conditioned supernatants of FaDu (**A**) and HeLa cells (**B**). Lower panels show representative images of HUVEC spheroids.

In addition to their ability to migrate, endothelial cells imperatively need to form sprouts so as to generate new vessels. Spheroids of HUVEC were grown *in vitro *and transferred into collagen gel along with a triple combination of WX-UK1, Galardin^®^, and Celecoxib^® ^or diluent only. After 24 hrs, the angiogenic potential of HUVEC spheroids was monitored as the ability to generate sprouts *in vitro *in collagen gel. HUVEC spheroids displayed a very low intrinsic capacity of sprouting in the absence of tumour cell supernatants (Figure 6, first column and picture). Addition of FaDu or HeLa conditioned supernatant to HUVEC spheroids induced the formation of 30 sprouts in average per spheroid. This capacity of tumour cell supernatants was almost abolished upon pre-treatment of cells with WX-UK1, Galardin^®^, and Celecoxib^® ^(Figure 6). Hence, the neo-angiogenic potential of FaDu and HeLa cells was strongly reduced upon inhibition of COX-2, MMPs, and the uPA-system.

## Discussion

One of the major drawbacks in anti-tumour therapy results from occult tumour cells that remain after surgery and/or radiochemotherapy. Owing to their invasion ability, these cells may lead to the formation of locoregional and/or distant metastases, which are associated with dramatically reduced overall survival times of tumour patients. The tumour cells are referred to as the origin of minimal residual disease (mrd) [24]. Whether MRD cells are equivalent to or bear similar properties as cancer-initiating cells [25], also referred to as cancer stem cells, is as to now unknown and under current investigation. Whatever the nature of MRD cells is, they require replicative, proteolytic, and neo-angiogenic potential to be the origin of secondary tumours and/or metastases [26]. As a consequence, multimodal therapeutic approaches promise to provide highest benefit for the afflicted patient. The extent of invasive capacity of tumour cells is very complex since different proteolytic cascades, enzymes, and cellular systems play crucial roles [27,28]. Malignant cells utilize the same arsenal of proteases to spread and invade as the healthy body for (patho)-physiological processes such as tissue remodelling, wound healing, traversal of immune cells through cell layers and embedding of the zygote in the uterine mucosa.

Accordingly, tumours hijack the normal control system of the proteases and use them for their own purposes. An excessive increase in the uPA system was shown to associate with tumour progression and metastasis formation [29-31], and an increase in MMPs is associated with degradation of ECM leading to the release of growth factors like bFGF and VEGF. This overexpression of MMP results is a predictor of a poor patient prognosis [32]. In addition to invasion and tissue remodelling, neo-angiogenesis is yet another major factor that influences tumour progression and metastasis formation [33]. Both, MMPs and COX-2 inhibitors have been well documented to have potent anti-angiogenetic effects [13,14,34,35]. As tumour biology is a complex mechanism, an effective therapy must consequently consist of a combination of various substances that inhibit different systems. In line with these findings, siRNA-mediated down-regulation of MMPs and members of the uPA system revealed instrumental. Targeting of those proteases in a combinatorial fashion inhibited invasion and angiogenesis [12]. Although novel concepts for the delivery of siRNA as therapeutic regimens are underway [11], alternatives, which *e.g. *include the use of chemical compounds as presented herein, are promising innovations. Furthermore, we expand the spectrum of malignancies to head and neck as well as cervix carcinoma lines.

Former studies using the serine protease inhibitor WX-UK1 were conducted with a spheroid confrontation model, in which the effects of WX-UK1 on the capacity of tumour cell spheroids to invaginate and invade fibroblast spheroids was tested. In average, tumour invasion was reduced by 50% upon treatment with WX-UK1 [17]. In the present study, invasion of single carcinoma cells in fibroblast spheroids was inhibited upon pre-treatment of tumour cells with combinations of the serine protease inhibitor WX-UK1, Galardin^®^, and Celecoxib^®^. This model reflects best the situation of MRD cells *in vivo*, which invade and remodel the surrounding tissue. Interestingly, significant inhibition using the MMP-inhibitor Galardin^® ^or the selective COX-2 inhibitor Celecoxib^® ^as single drugs was not observed and is in discordance with the pervading literature [36,37] describing an inhibition of invasion by 40% after Celecoxib^® ^treatment. A possible explanation for this discrepancy is the use of YD-10B cells, which might reveal more responsive to Celecoxib^® ^than FaDu and HeLa cells as used in the present study. Another study demonstrated that the invasion reduction upon COX-2 inhibitors also appears to act independently of COX-2 [38-40], potentially explaining the discrepancy in inhibition despite similar COX-2 levels. Most importantly, treatment of tumour cells with the triple combination displayed a synergistic effect: inhibition of the invasion of FaDu and HeLa cells was in any case above 80% and peaked at 95%. Hence, the usage of WX-UK1, Galardin^®^, and Celecoxib^® ^as a combinatorial therapy is conceivable.

The triple therapy has not only ascendancy on invasion but also inhibits angiogenesis. The distribution of growth factors such as Vascular endothelial growth factor (VEGF) or basic fibroblast growth factor (bFGF) advocates the migration of endothelial cells as well as the sprouting of new blood vessels, allowing the tumour to establish contact with the blood system and subsequently increase metastasis. In the same manner, the production of bFGF was decreased through the use of all single, double and triple therapy combinations by 60-80%, but without an additional or synergistic effect. These data are in line with Hasegawa *et al. *[41] and Basu *et al. *[42] showing an inhibition of bFGF and VEGF using a selective COX-2 inhibitor. Treatment of tumour cells with the triple combination of inhibitors not only impacted on bFGF release but also displayed functional effects. The release of chemoattractants by FaDu and HeLa cells was substantially reduced upon treatment, and resulted in diminished migratory capacity of HUVEC towards tumour cell supernatant and reduced tube formation. Again, these results are in full accordance with Basu *et al.*, who pointed out that Celecoxib reduced the formation of blood vessels [42].

Taken together, the combination of different inhibitors appears as a promising concept in multimodal tumour therapy, particularly because tumour cells often develop resistances against single therapies.

## Conclusions

Combinatorial treatment of carcinoma cells with inhibitors of the uPA system (WX-UK1), MMPs (Galardin), and COX-2 (Celecoxib) is superior to the single treatment with WK-UK1 alone. Triple medication inhibited the invasive and angiogenic potential of carcinoma cells by 90% and 70%, respectively. Hence, multimodal therapy involving the presented targets is a promising approach to treat cancer.

## Competing interests

Bernd Mühlenweg is a full-time employee of Wilex AG.

## Authors' contributions

**PZ **performed and coordinated parts of the experiments, wrote the manuscript, and analysed the data. **DR **and **BM **performed experiments. **SZ **provide help with angiogenesis experiments. **AB **helped organising and correcting the manuscript. **BMue **provided help with WX-UK1. **OG **coordinated the work, analysed the data, and wrote the manuscript. **SS **performed experiments and coordinated parts of the work. All authors read and approved the final manuscript.

## Pre-publication history

The pre-publication history for this paper can be accessed here:

http://www.biomedcentral.com/1471-2407/10/92/prepub

## Supplementary Material

Additional file 1 **Effects of chemical inhibitors on cell vitality**. The effects of Galardin (MMPI), WX-UK1 (SPI), and COX2 inhibitor (COXI) were assessed in standard MTT assays at maximal tolerable doses of each compound and in the combinations indicated. Shown are the mean and standard deviations of three independent experiments. Click here for file
