# Supplementary figures and images for: Multimodal therapy for synergic inhibition of tumour cell invasion and tumour-induced angiogenesis
Source: BMC Cancer. 2010 Mar 11;10:92. doi: 10.1186/1471-2407-10-92 (PMC2841144; doi:10.1186/1471-2407-10-92)

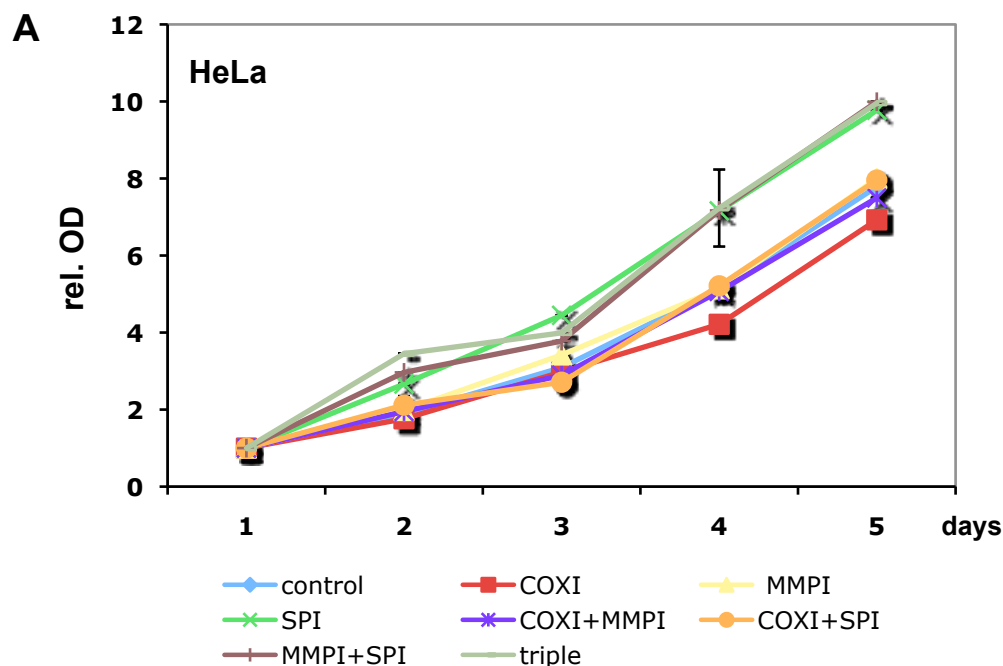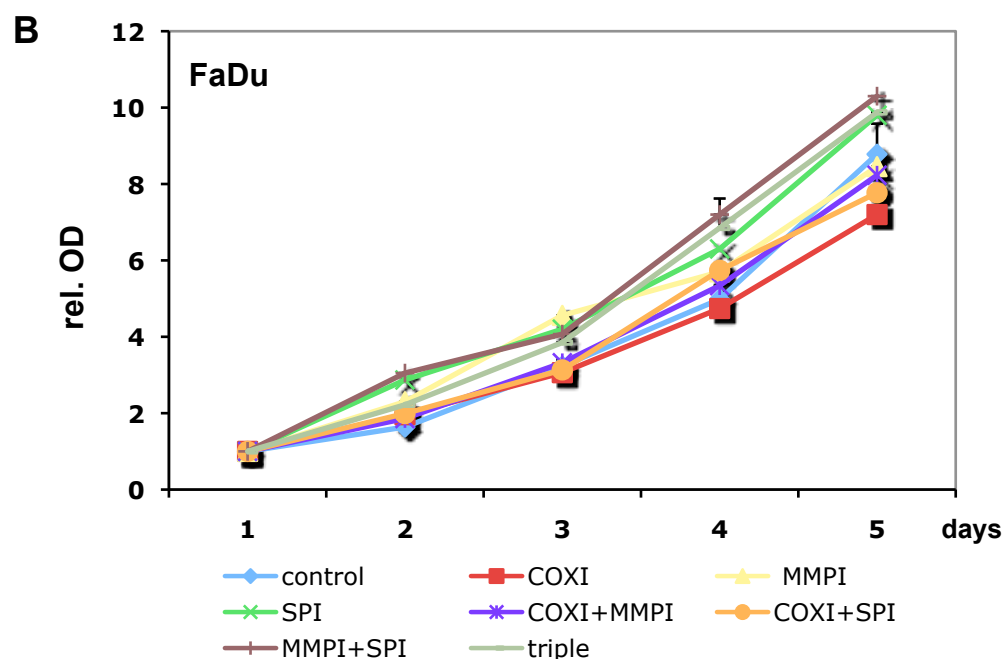

**Supplementary Figure 1**  
**Zengel *et al* (2009)**

Supplement: Additional file 1 — Effects of chemical inhibitors on cell vitality. The effects of Galardin (MMPI), WX-UK1 (SPI), and COX2 inhibitor (COXI) were assessed in standard MTT assays at maximal tolerable doses of each compound and in the combinations indicated. Shown are the mean and standard deviations of three independent experiments. [file 1471-2407-10-92-S1.PDF]
